# A five‐CpG signature of microRNA methylation in non‐G‐CIMP glioblastoma

**DOI:** 10.1111/cns.13133

**Published:** 2019-04-23

**Authors:** En‐Ming Kang, An‐An Yin, Ya‐Long He, Wei‐Jun Chen, Amandine Etcheverry, Marc Aubry, Jill Barnholtz‐Sloan, Jean Mosser, Wei Zhang, Xiang Zhang

**Affiliations:** ^1^ Department of Neurosurgery, Xijing Institute of Clinical Neuroscience, Xijing Hospital Air Force Medical University Xi'an China; ^2^ Department of Neurosurgery The 88th Hospital of the People's Liberation Army Taian China; ^3^ Department of Emergency Medicine, Jinling Hospital Medical School of Nanjing University Nanjing China; ^4^ CNRS, UMR 6290 Institut de Génétique et Développement de Rennes (IGdR) Rennes France; ^5^ UEB, UMS 3480 Biosit, Faculté de Médecine Université Rennes1 Rennes France; ^6^ CHU Rennes Service de Génétique Moléculaire et Génomique Rennes France; ^7^ Plate‐forme Génomique Santé Biosit Université Rennes1 Rennes France; ^8^ Case Comprehensive Cancer Center Case Western Reserve University Cleveland Ohio

**Keywords:** angiogenesis, DNA methylation signature, glioblastoma, miRNA, prognostication

## Abstract

**Aims:**

DNA methylation has been found to regulate microRNAs (miRNAs) expression, but the prognostic value of miRNA‐related DNA methylation aberration remained largely elusive in cancers including glioblastomas (GBMs). This study aimed to investigate the clinical and biological feature of miRNA methylation in GBMs of non‐glioma‐CpG island methylator phenotype (non‐G‐CIMP).

**Methods:**

Prognostic miRNA methylation loci were analyzed, with TCGA and Rennes cohort as training sets, and independent datasets of GBMs and low‐grade gliomas (LGGs) were obtained as validation sets. Different statistical and bioinformatic analysis and experimental validations were performed to clinically and biologically characterize the signature.

**Results:**

We identified and validated a risk score based on methylation status of five miRNA‐associated CpGs which could predict survival of GBM patients in a series of training and validation sets. This signature was independent of age and O‐6‐methylguanine‐DNA methyltransferase (*MGMT*) promoter methylation status. The risk subgroup was associated with angiogenesis and accordingly differential responses to bevacizumab‐contained therapy. MiRNA target analysis and in vitro experiments further confirmed the accuracy of this signature.

**Conclusion:**

The five‐CpG signature of miRNA methylation was biologically relevant and was of potential prognostic and predictive value for GBMs. It might be of help for improving individualized treatment.

## 1 INTRODUCTION

Glioblastomas (GBMs) are the most common and devastating subtypes of primary central nervous system tumors.^1^ Unfortunately, despite the multimodal treatment of surgical resection, radiotherapy, and chemotherapy, the reported median survivals of GBM patients were only 16‐19 months.^1^, ^2^, ^3^

Cancer‐specific DNA methylation changes play important roles in cancer development and progression. The best‐known epigenetic abnormality in cancers is promoter‐specific CpG island (CGI) hypermethylation of tumor suppressor genes which consequently cause transcriptional silencing.^4^ Altered DNA methylation affected the expressions of not only protein‐coding genes but also noncoding RNAs (ncRNAs).^5^ Among those ncRNAs, microRNAs (miRNAs), the 20‐22 nucleotides small ncRNAs, have been demonstrated to have multiple roles in the pathogenesis of cancers.^6^ It has been reported that miRNAs could be regulated by DNA methylation and abnormal methylation in miRNAs was closely correlated with cancer progression.^6^, ^7^ However, the biological and clinical implications of miRNA methylation abnormality were largely unstudied in cancers including GBMs. Glioma‐CpG island methylator phenotype (G‐CIMP) represents a distinct subgroup of glioma which is featured by genome‐wide hypermethylated CGIs and favorable prognosis.^8^ The G‐CIMP+ tumors have already been broadly studied, while the relevance features of non‐G‐CIMP GBMs remain largely unclear.

In this study, we analyzed miRNA methylation data of non‐G‐CIMP GBMs from The Cancer Genome Atlas (TCGA), Gene Expression Omnibus (GEO), and Rennes cohort^9^ to reveal the relationship between miRNA methylation and GBM survival. Bioinformatic methods and in vitro experiments were used to validate our results.

## 2 MATERIALS AND METHODS

### 2.1 GBM datasets

Rennes cohort of 77 newly diagnosed non‐G‐CIMP GBMs with clinical and genome‐wide DNA methylation microarray data by Infinium HumanMethylation450k BeadChip (Illumina Inc, San Diego, CA, USA) was obtained from the ArrayExpress under the accession number “E‐MTAB‐4969.”^9^ All patients received standard adjuvant treatment of radiotherapy (RT) and concurrent temozolomide (TMZ). Public DNA methylation datasets of non‐G‐CIMP GBM samples were also downloaded from The Cancer Genome Atlas (TCGA) data portal,^10^ and Gene Expression Omnibus (GEO) under the accession number “GSE60274.”^11^ ( Detailed clinical data of and relative CpG information are listed in the Supporting Information S1）We also obtained clinical and DNA methylation data of LGGs from TCGA^12^ and GSE48462.^13^ Among the heterogeneous datasets, only those with age over 18 years old and a molecular diagnosis of non‐G‐CIMP tumors were included in this study. For survival analysis, patients with a follow‐up data >1 month were included, in order to reduce the bias caused by noncancer death.^10^ In addition, nontumor brain tissues were obtained from apparently healthy individuals or chronic epilepsy patients with pathological evidence of other neurological or psychiatric diseases in each dataset. The G‐CIMP status was determined by K‐means (*k* = 3) clustering on the 1503 probes reported by Noushmehr et al^14^ *MGMT* (O‐6‐methylguanine‐DNA methyltransferase) promoter methylation status was determined by a logistic regression model using two CpGs, that is, cg12434587 and cg12981137.^15^ Batch effects from different datasets and platforms were adjusted by a nonparametric empirical Bayes approach (*ber* package).^16^ Methylation level of each integrated CpGs was summarized as *M*‐value.^17^

### 2.2 Construction and validation of a miRNA methylation‐based risk score model

CpG probes were filtered by removing those targeting the X and Y chromosomes, containing a single nucleotide polymorphism (SNP) within five base pairs of the targeted CpG. We then selected probes annotated with miRNAs (n = 2448) for this study. The discovery phase was performed within TCGA and Rennes cohort (training sets). Univariate Cox regression analysis with permutation test was performed within each training set. Potential prognostic CpGs with consistent survival correlation (permutation *P* < 0.2) in each training set were subjected to multivariate Cox regression analysis within the combined training set (TCGA and Rennes collectively), and those with a *P* value < 0.05 were finally selected for risk score modeling. The risk score formula was constructed by integrating the *M*‐values of all selected CpGs which were weighted by their multivariate Cox regression coefficients after adjusted by patient age and *MGMT* promoter methylation status.^18^, ^19^ Patients were then classified into high‐risk or low‐risk groups with the cutoff point as the median risk score from the combined training set. The validation phase was performed in GSE60274 and datasets of LGGs and in particular those with wide‐type IDH.

### 2.3 Gene Set Enrichment Analysis (GSEA)

GSEA was performed to evaluate the functional gene expression profiles between the risk subgroups on reported gene sets from Molecular Signature Database (MSigDB), with nominal *P* value ≤ 0.05 for significance.^20^

### 2.4 MiRNA target gene prediction and pathway analysis

The online databases TargetScan (http://www.targetscan.org/vert_72/. Accessed November 11, 2018), miRanda (http://www.microrna.org/microrna/home.do. Last update: 2010‐11‐01), and miRDB (http://mirdb.org/. Accessed November 11, 2018) were used to identify the target genes of the relative miRNAs. Genes appeared in all three databases were included for the following analysis.^21^ GO analysis was then performed with PANTHER (version 14.0 Released 2018‐12‐03) based on the GO database (http://www.geneontology.org/ Accessed January 11, 2019) for biological process (BP), cellular component (CC), molecular function (MF), and pathway enrichment of the predicted target genes.^22^ Fisher's two‐side exact test was used to classify the GO categories, and the Bonferroni correction for multiple test was calculated to correct the *P* values. Bonferroni‐corrected for *P* < 0.05 was considered to be significant. Enrichment analysis based on Kyoto Encyclopedia of Genes and Genomes (KEGG) was performed and visualized using ClueGO (Version 2.5.3),^23^, ^24^ a Cytoscape (version 3.7.1, http://cytoscape.org/) plug‐in. The main parameters for constructing the network were as follows: ontologies/pathways: KEGG (321 terms/pathways with 7454 available unique genes, December 7, 2018), GO term/pathway selection: Min Gene = 3 & Min Percentage = 3.0%, Kappa Score Threshold = 0.5, Statistical Test Used = Enrichment/Depletion (Two‐sided hypergeometric test), Correction Method Used = Bonferroni step down. Only pathways with *P* value < 0.05 were considered to be significant.

### 2.5 Cell culture and transfection

The human GBM cell lines U87MG, U251, T98G, and SHG44 were obtained from the cell bank of the Air Force Medical University (Xi'an, China) and cultured in Dulbecco's modified Eagle's medium (DMEM; Gibco, USA) supplemented with 10% fetal bovine serum (FBS; Shanghai BioSun Sci&Tech Co., Ltd., Shanghai, China) and incubated at 37°C with 5% CO_2_. MiR‐1284 mimic (UCU AUA CAG ACC CUG GCU UUU C) and mimic negative control (mimic NC; UUC UCC GAA CGU GUC ACG UTT) were synthesized by Sangon Biotech Co., Ltd. (Shanghai, China). Cells for transfection were planted into 60‐mm dishes at 4 × 10^5^ cells/well. After 48 hours incubation, miR‐1284 mimic, mimic NC, or control (only treated with Polymer) was transfected into cells at 50 pmol/mL using Xfect RNA Transfection Reagent (Takara Bio, USA). The transfection efficiency was verified by real‐time quantitative PCR (qPCR).

### 2.6 RNA extraction and Real‐time quantitative PCR

Total RNA was extracted by TRIzol reagent (Invitrogen, USA) and reverse transcribed with PrimeScript RT reagent kit (Takara, Tokyo, Japan). PCR amplification was performed in triplicate with SYBR Premix Ex Taq II (Takara) using CFX96 Real‐Time PCR Detection System (Bio‐Rad, Hercules, CA, USA). The expression values of miR‐1284 were normalized to the levels of small nuclear U6. The primer sequences were listed as follows: (a) miR‐1284: Reverse transcription primer: 5′‐CTC AAC TGG TGT CGT GGA GTC GGC AAT TCA GTT GAG GAA AAG‐3′; (b) U6 Reverse transcription primer: 5′‐CGC TTC ACG AAT TTG CGT GTC AT‐3′; miR‐1284‐F: 5′‐CGT CTA TAC AGA CCC TGG CTT TTC‐3′; miR‐1284‐R: 5′‐CTC AAC TGG TGT CGT GGA‐3′; U6‐F: 5′‐CTC GCT TCG GCA GCA CAT A‐3′; U6‐R: 5′‐CGC TTC ACG AAT TTG CGT G‐3′.

### 2.7 Pyrosequencing

Pyrosequencing was performed by Pyromark Q96 ID platform and analyzed by PyroMark CpG software (Qiagen, Germany). The following primers were used: miR‐1284‐F 5′‐ATT TTT ATT GGT TAA ATT AAT ATT ATA GG‐3′, miR‐1284‐R biotin‐5′‐AAC TTA TTA CAT TAA ATA CAA ACA ACA AC‐3′, miR‐1284‐seq 5′‐TTT TTA GTT TTT AAG TAT ATT‐3′. The DNA methylation value for each sample was calculated as the average methylation value of the interrogated CpGs.^25^

### 2.8 5‐Aza‐2′‐deoxycytidine (5‐Aza‐dC) demethylation treatment

U251 and U87MG cells were grown for 4 days in the presence of 10 μmol/L 5‐Aza‐dC (Sigma‐Aldrich, St. Louis, MO, USA). Fresh 5‐Aza‐dC was added every 24 hours.

### 2.9 Cell proliferation assay

Cells with different treatments were implanted in 96‐well plates at 5 × 10^3^ per well. At indicated time points, CCK‐8 kit (Yeasen, Shanghai, China) was assayed for cell viability measurement.

### 2.10 Cell cycle and apoptosis analysis

For cell cycle analysis, cells were harvested, fixed in 70% ethanol on ice, and stained with propidium iodide in phosphate‐buffered saline containing RNase. The DNA contents were analyzed by flow cytometry. For cell apoptosis analysis, Annexin V‐fluorescein isothiocyanate and propidium iodide double staining (Roche Diagnostics, Germany) was used to sort cells in early or late apoptotic phase.

### 2.11 Wound‐healing assay

Cell motility was assessed by wound‐healing assay as described previously.^26^ A scratch wound was generated by a 200 μL pipette tip on the confluent cell monolayers in 6‐well plates. The spread of the wound closure was observed after 48 hours of the scratch.

### 2.12 Statistical analysis

The distributions of known molecular and clinical features with respect to the risk subgroups were tested by Fisher's exact or chi‐square test. Survival data, for example, overall survival (OS) and progression‐free survival (PFS), were estimated by the Kaplan‐Meier method and compared by log‐rank test. Univariate and multivariate Cox regression models were performed to evaluate the correlation and independence of potential prognostic factors. For in vitro experiments, data were expressed as mean ± SEM from three independent experiments and analyzed by Student's *t* test. All the calculations were done within SPSS19.0 (IBM Corporation, New York, NY, USA) and R software (version 3.2.5; https://www.r-project.org/), and a difference was considered significant when *P* ≤ 0.05.

## 3 RESULTS

### 3.1 Identification of prognostic miRNA methylation loci from the training sets

**Figure 1:**
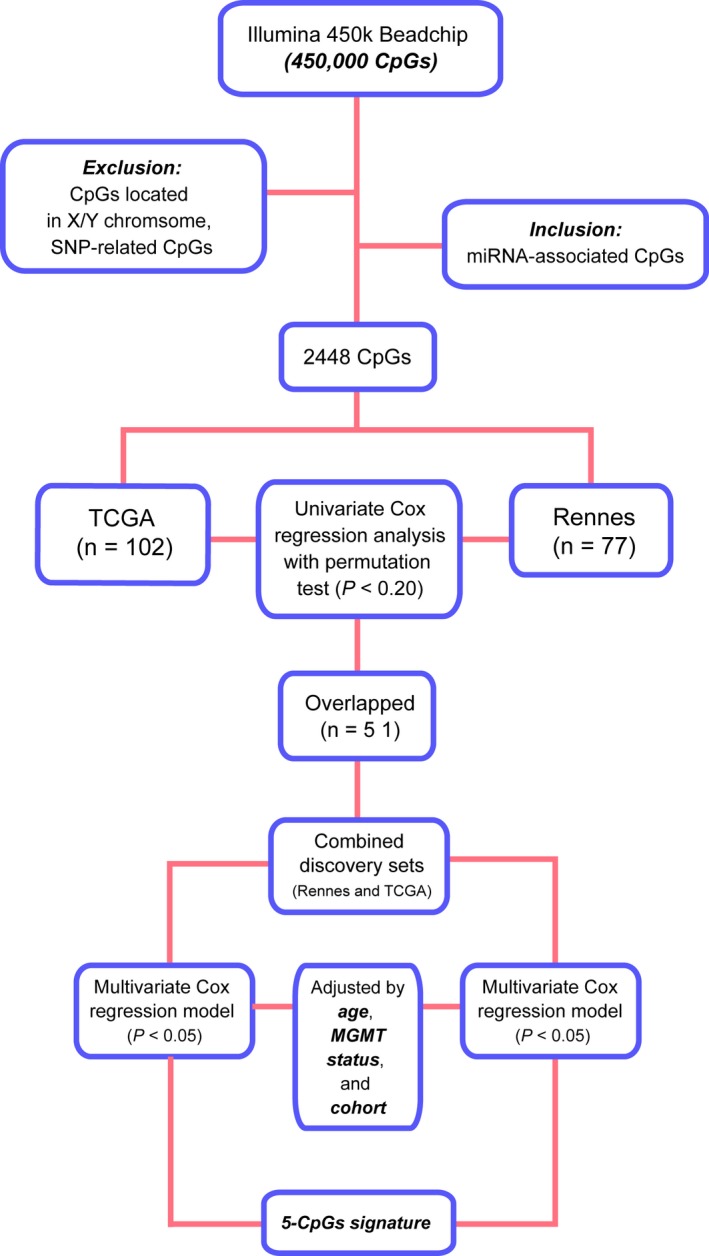
Schematic diagram of the probe selection workflow for the study

The included cohorts and the workflow of probe selection were schematically presented in Figure 1, and patient characteristics were summarized in Table 1. By employing a multistep selection criterion, we identified a five‐CpG panel of miRNA methylation that showed consistent prognostic significance in both training sets (Table 2). Among the panel, two CpGs (eg, cg05744073 and cg08244382) were hypermethylated and one CpG (eg, cg13767001) was hypomethylated in GBMs, while the other two were not differentially methylated in GBMs (Table 2). Upon the correlation with prognosis, three CpGs (eg, cg05744073, cg08244382, and cg20382675) showed negative correlation with OS, while two CpGs (eg, cg24082174 and cg13767001) with positive correlation (Table 2).

**Table 1:** Patient characteristics of included patient cohorts of non‐G‐CIMP GBMs

Variables	Training set		Validation set
	Rennes cohort	TCGA	GSE60274
Sample size	77	102	59
Clinical factors			
Age			
Median	60	63	52
Range	36‐75	23‐85	26‐70
Pre‐operative KPS^a^			
Median	80	80	NA
Range	40‐100	40‐100	NA
Gender			
Male/Female	55/22	58/44	45/14
Extent of surgery			
Surgery (total/partial)/Biopsy	72 (55/17)/4	101 (NA/NA)/1	57 (NA/NA)/2
Adjuvant Treatments			
RT + TMZ/RT	77/0	71/31	32/27
BVZ/non‐BVZ/UN	29/32/16	NA	NA
Molecular factors			
*MGMT* methylation status			
Methylated/Unmethylated	26/51	37/65	26/33
Gene expression subtype			
P/N/C/M	18/6/24/27	20/13/37/30	8/4/17/20
TCGA methylation clusters			
Clusters 2/3	29/48	35/67	23/36

KPS, Karnofsky performance score; NA, not available; RT, radiotherapy; TMZ, temozolomide; UN, unknown.

a KPS was available for only a small subset of patients from TCGA cohort.

**Table 2:** The five prognostic CpGs associated with miRNA

Probes	Chr.	miRNA name	miRNA region	Relation to CpG island	Methylation status in GBM	Average *M*‐value of high‐risk GBMs^a^	Cox regression coefficients
cg05744073	17	miR‐132	Body	Island	Hypermethylated	−4.073	−0.534
cg08244382	14	miR‐127; miR‐433	TSS1500;TSS200	Island Shore	Hypermethylated	3.185	−0.446
cg20382675	3	miR‐1284	TSS200	Open sea	NS	0.287	−0.263
cg24082174	3	miR‐1248	TSS1500	Island Shore	NS	0.991	0.255
cg13767001	13	miR‐759	TSS1500	Open sea	Hypomethylated	−2.223	0.368

NS, no significance; TSS, transcription start sites.

Methylation level assessed with *M*‐value: low (‐Inf, −2), middle [−2, 2], and high (2, Inf).

a Included all high‐risk samples of three datasets.

**Figure 2:**
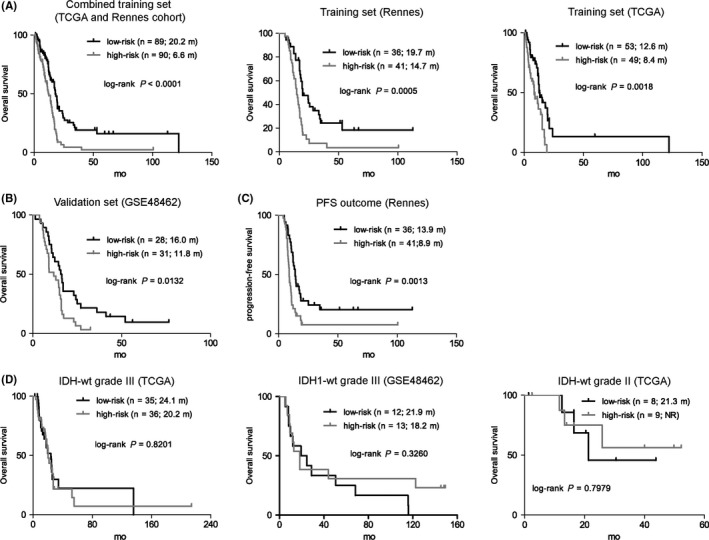
The survival correlation of the five‐CpG signature in each dataset. A, The five‐CpG signature predicted overall survival (OS) in training sets. B, The signature was validated by yielding apparent OS difference in GSE60274. C, The five‐CpG signature was also able to predict PFS in Rennes cohort. D, The signature could not identify patients with different prognoses in IDH wide‐type LGG (grade III or II)

Accordingly, the risk score model was constructed as follows: risk score = (−0.534 × *M*‐value of cg05744073) + (−0.446 × *M*‐value of cg08244382) + (−0.263 × *M*‐value of cg20382675) + (0.254 × *M*‐value of cg24082174) + (0.368 × *M*‐value of cg13767001). With the cutoff as the median risk score from the combined training sets (−0.382), patients were divided into low‐risk groups (with lower risk scores) and high‐risk groups (with higher risk scores). In the combined training sets, the assigned low‐risk patients (n = 89) were significantly associated with longer overall survival (OS) than those high‐risk ones (n = 90; log‐rank *P* < 0.0001; Figure 2A). The 5‐CpG signature also showed consistent prognostic value in each training set (Figure 2A).

### 3.2 Validation of the five‐GpG miRNA methylation signature for prognostication

To validate the prognostic performance of the 5‐CpG miRNA methylation signature, we applied it to the independent validation set of GSE60274. With the prespecified cutoff, patients were classified into a low‐risk group (n = 28) and a high‐risk group (n = 31). Consistent with the training sets, low‐risk patients were associated with longer OS than high‐risk ones (log‐rank *P* = 0.013; Figure 2B). We also observed a significant correlation between progression‐free survival (PFS) and the miRNA methylation‐based risk groups in Rennes cohort (Figure 2C).

In addition, we applied the GBM‐derived signature to independent validation cohorts of IDH wild‐type LGGs. The miRNA methylation signature failed to yield significant OS differences between the risk subgroups within both grade III and II gliomas using their median risk scores as cutoffs, respectively, which supported the signature as a GBM‐specific prognostic model (Figure 2D).

### 3.3 Molecular and clinical correlation of the 5‐CpG miRNA methylation signature

**Figure 3:**
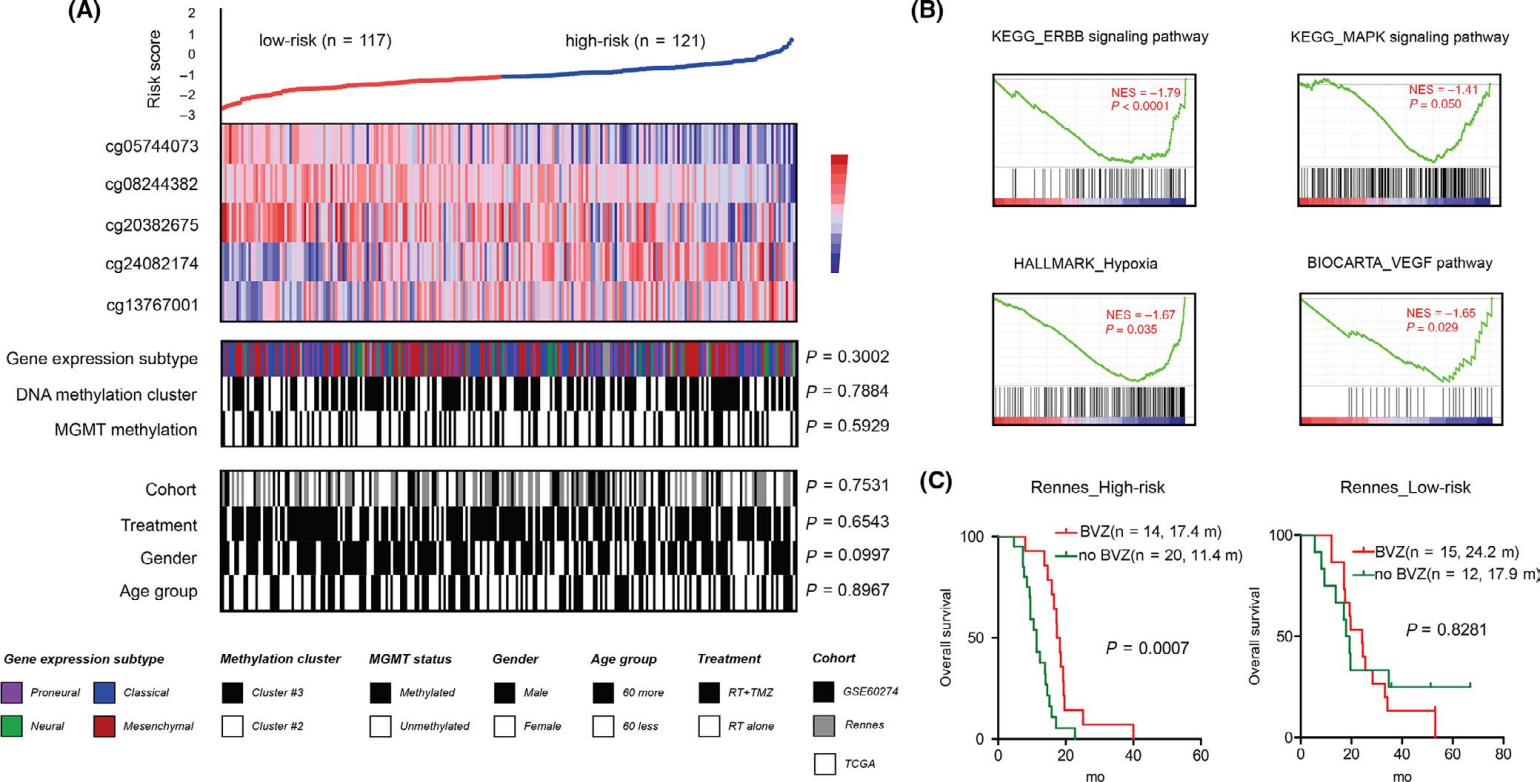
Molecular and clinical characteristics of the 5‐CpGs miRNA methylation signature. A, the heat maps of K‐means (*k* = 2) clustering on the 5‐CpGs methylation signature according to the *M*‐value from all GBM groups; each column represented a sample; for each sample (n = 238), subgroup correlation was indicated; *P* values for Fisher' exact test and chi‐square test were accordingly shown; B, GSEA enrichment plots for representative functional gene sets enriched in high‐risk tumors from TCGA. C, High‐risk but not low‐risk tumors conferred significant OS benefits when treated with bevacizumab in Rennes cohort with available second‐line therapies

Correlation with current established molecular features showed that the 5‐CpG signature appeared not to be significantly correlated with TCGA gene expression subtypes, DNA methylation clusters, and *MGMT* promoter methylation status (Figure 3A). Also, the signature seemed not to be correlated with treatments, gender, and age (Figure 3A). GSEA on gene expression data of TCGA samples showed that the high‐risk tumors were enriched with pro‐oncogenic gene sets such as ErbB signaling pathway (*P* < 0.0001), MAPK signaling pathway (*P* = 0.029), pro‐angiogenic gene sets such as hypoxia (*P* = 0.035), and VEGF pathway (*P* = 0.029), which might biologically explain the inferior survival of those high‐risk tumors (Figure 3B).

### 3.4 High‐risk patients appeared to be beneficial for bevacizumab therapy

As reported by GSEA, high‐risk tumors seemed to be featured by upregulation of various pro‐angiogenic gene sets (Figure 3B). Accordingly, we tested the potential survival benefits conferred by the anti‐angiogenic agent bevacizumab as combined therapy within each risk subgroup. In Rennes cohort with available second‐line therapies, we found that the addition of bevacizumab did confer significant OS benefits in high‐risk tumors, but was associated with similar OS to bevacizumab‐free therapy (Figure 3C).

### 3.5 The 5‐CpG signature was an independent prognostic factor in non‐G‐CIMP GBMs

Within Rennes cohort with RT/TMZ, univariate Cox regression analysis showed that patient age, *MGMT* promoter methylation status, and our miRNA methylation signature were significantly associated with OS (Table 3). Multivariate Cox regression analysis further demonstrated the prognostic independence of the abovementioned factors (Table 3). Multivariate Cox regression model within the combined cohorts of TCGA, GSE60274, and Rennes cohort not only confirmed the prognostic independence of our miRNA methylation signature but also supported its treatment independence (Table 3).

**Table 3:** Results of the miRNA methylation signature in Cox regression analysis

Variables	Univariate Cox model			Multivariate Cox model		
	HR	95% CI	*P* value	HR	95% CI	*P* value
Rennes (n = 61)^a^						
Patient age	1.046	1.015‐1.078	**0.003**	1.040	1.003‐1.078	**0.033**
miRNA methylation signature	2.926	1.733‐4.942	**<0.001**	3.129	1.782‐5.493	**<0.001**
*MGMT* methylation status	0.438	0.236‐0.813	**0.009**	3.047	0.140‐0.569	**<0.001**
*DNA methylation clusters*	0.849	0.492‐1.465	0.557			
Proneural subtype	0.905	0.483‐1.695	0.754			
BVZ treatment	0.607	0.357‐1.031	0.065	0.536	0.273‐1.049	0.069
Gender	0.918	0.522‐1.614	0.767			
Extent of surgery	0.957	0.623‐1.469	0.840			
TCGA + GSE60274 + Rennes (n = 238)^b^						
Patient age	1.028	1.012‐1.044	**0.001**	1.034	1.018‐1.051	**<0.001**
Treatments (RT/TMZ vs RT)	0.479	0.345‐0.666	**<0.001**	0.438	0.314‐0.609	**<0.001**
*DNM methylation clusters*	0.995	0.732‐1.351	0.973			
miRNA methylation signature	2.207	1.704‐2.859	**<0.001**	2.368	1.838‐3.050	**<0.001**
*MGMT* methylation status	0.627	0.455‐0.863	**0.004**	0.589	0.427‐0.812	**0.001**
Gender	1.009	0.732‐1.392	0.956			

KPS, Karnofsky performance score; NA, not available; RT, radiotherapy; TMZ, temozolomide.

^a^ Rennes cohort excluded 16 patients with insufficient treatment information.

^b^ Including all patients from TCGA, Rennes cohort, and GSE60274.

^c^ The significance of bold values indicate *P* value < 0.05.

### 3.6 The prognostic value of the miRNA methylation signature with respect to current GBM classification

**Figure 4:**
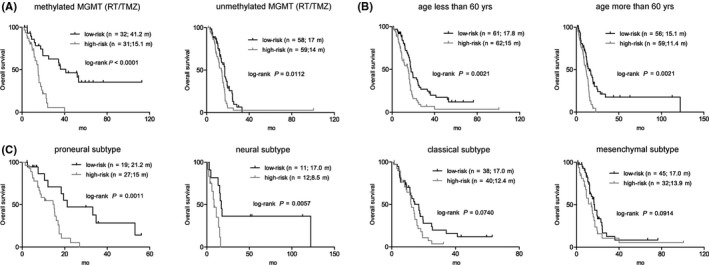
The survival correlation of the five‐CpG signature within current GBM classification. A, The five‐CpG signature predicted overall survival (OS) in both MGMT promoter methylated and unmethylated patients treated with both radiotherapy (RT) and temozolomide (TMZ). B, It was also correlated with different OS in subgroups of ≤60 or ＞60 y. C, The correlation between five‐CpG signature and different prognoses was significant in proneural and neural subtypes and marginally significant in the classical and mesenchymal subtypes

We also tested the prognostic interrelationship of the 5‐CpG signature with known prognostic factors within available patients from the combined training and validation sets. We found that it could consistently predict OS within the subtypes of unmethylated or methylated *MGMT* tumors (Figure 4A), and the subgroups of ≤60 or＞60 years (Figure 4B). Regarding the TCGA expression subtypes, the signature was significantly associated with OS in the proneural and neural subtypes, and also yielded marginally significant OS difference in the classical and mesenchymal subtypes (Figure 4C).

### 3.7 Target gene prediction of the 5‐CpG signature‐related miRNAs

**Figure 5:**
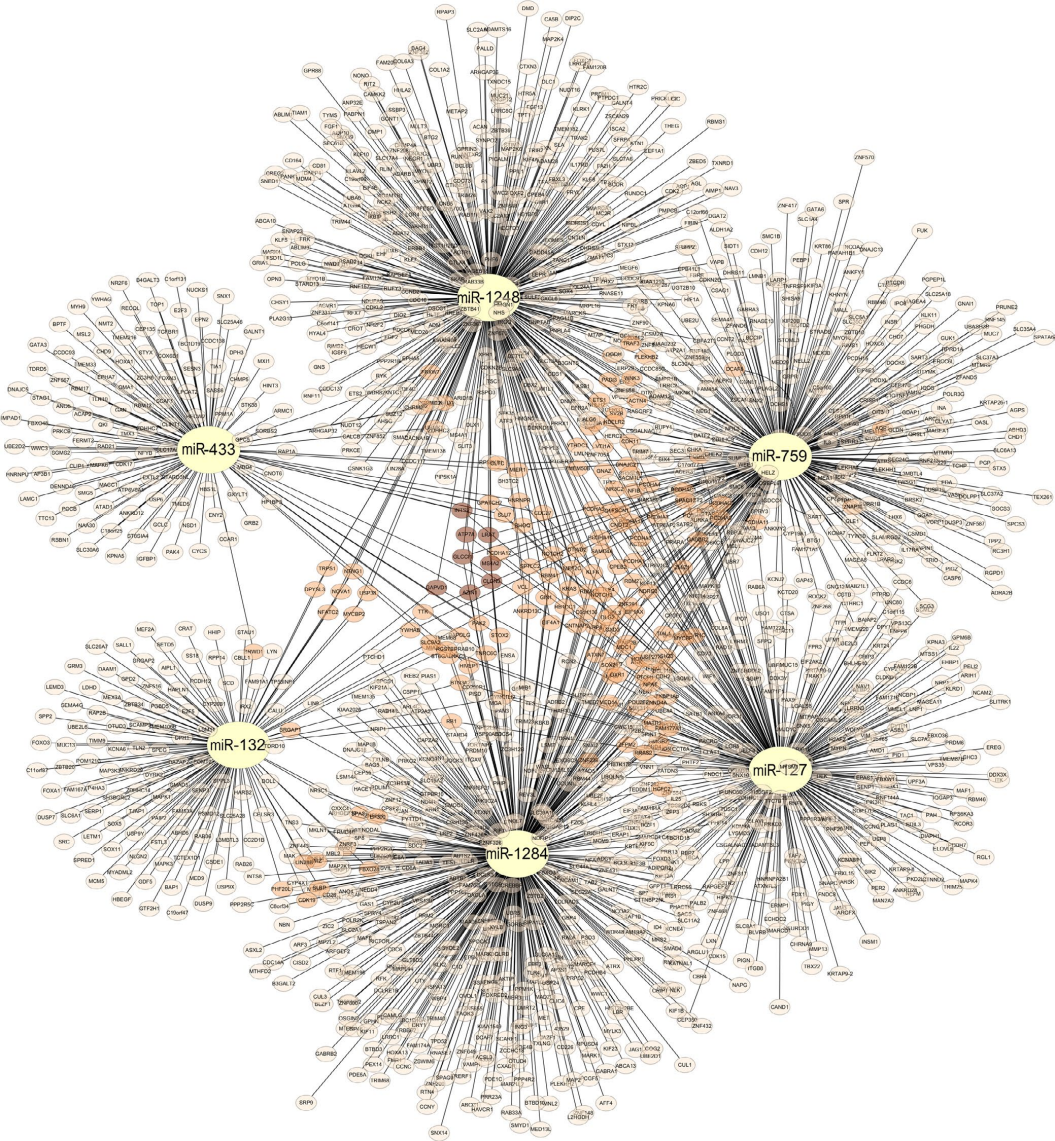
Target prediction results of signature associated miRNAs

Targetscan, miRanda, and miRDB databases were used to predict the target genes of miR‐132, miR‐127, miR‐433, miR‐1284, miR‐1248, and miR‐759. To ensure the specificity and sensitivity of our prediction, we kept the identical targets predicted in all three databases without setting additional criterion. Totally 1578 target genes left for further functional analysis (Figure 5). Among them, 139 genes were candidate targets for two miRNAs, seven genes were common targets of three miRNAs, and one was target of four miRNAs.

### 3.8 Biological characteristics of predicted target genes

**Figure 6:**
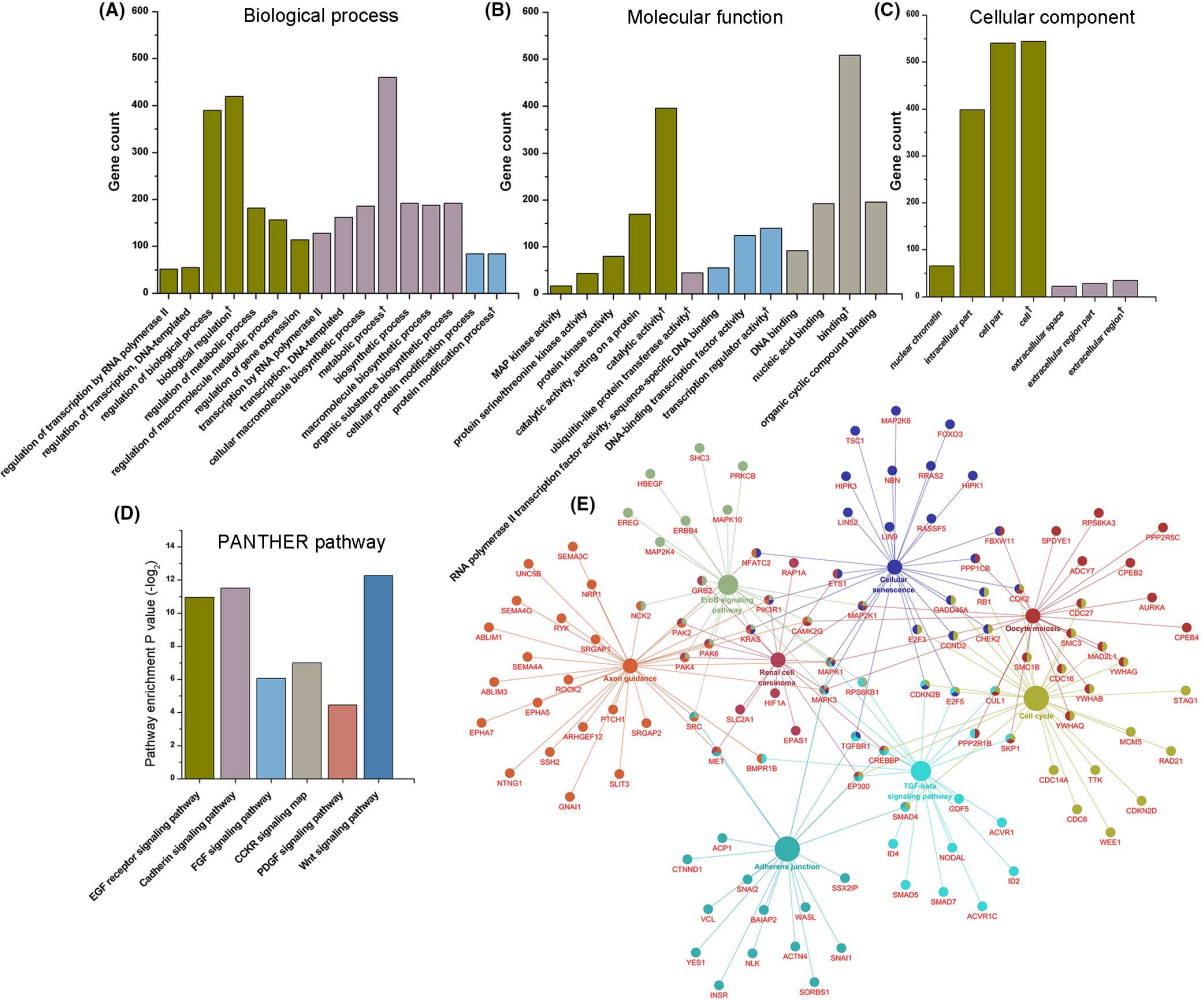
Bioinformatic analysis of predicted target genes. A, PANTHER GO‐Slim biological process. B, PANTHER GO‐Slim molecular function. C, PANTHER GO‐Slim cellular component. D, PANTHER pathway enrichment. E, KEGG pathway enrichment analysis, relative genes were shown as well

Predicted target genes were further analyzed using PANTHER GO‐slim tools on MF, BP, and CC (Table 4). The most enriched MF terms were MAP kinase activity, ubiquitin‐like protein transferase activity, DNA binding, and RNA polymerase II transcription factor activity (Figure 6A). The most enriched BP terms were regulation of transcription by RNA polymerase II, transcription by RNA polymerase II, and cellular protein modification process (Figure 6B). The most enriched CC terms were nuclear chromatin and extracellular space (Figure 6C). PANTHER pathway analysis showed EGF receptor signaling pathway, cadherin signaling pathway, FGF signaling pathway, CCKR signaling pathway, PDGF signaling pathway, and Wnt signaling pathway were the most enriched pathways (Figure 6D). Then, we utilized ClueGO to make a KEGG pathway enrichment analysis (Figure 6E, Table 5). The most enrichment terms were adherens junction, cell cycle, TGF‐beta signaling pathway, ErbB signaling pathway, axon guidance, renal cell carcinoma, oocyte meiosis, and cellular senescence.

**Table 4:** PANTHER analysis for predicted target genes

Terms	Target gene	Expected gene Nr	Fold enrichment	*P* value
PANTHER GO‐slim molecular function				
MAP kinase activity	17	5.04	3.38	0.028
→Protein serine/threonine kinase activity	44	20.45	2.15	0.005
→Protein kinase activity	80	38.61	2.07	<0.001
→Catalytic activity, acting on a protein	170	99.8	1.7	<0.001
→Catalytic activity	396	324.05	1.22	0.012
Ubiquitin‐like protein transferase activity	45	22.2	2.03	0.017
RNA polymerase II transcription factor activity, sequence‐specific DNA binding	56	27.85	2.01	0.002
→DNA‐binding transcription factor activity	125	75	1.67	<0.001
→Transcription regulator activity	140	83.93	1.67	<0.001
DNA binding	92	56.39	1.63	0.008
→Nucleic acid binding	193	125.21	1.54	<0.001
→Binding	509	404.85	1.26	<0.001
→Organic cyclic compound binding	196	129.71	1.51	<0.001
Unclassified	738	859.06	0.86	0
PANTHER GO‐slim biological process				
Regulation of transcription by RNA polymerase II	52	26.71	1.95	0.044
→Regulation of transcription, DNA‐templated	55	28.84	1.91	0.038
→regulation of biological process	390	287.8	1.36	<0.001
→Biological regulation	420	312.6	1.34	<0.001
→Regulation of metabolic process	182	106.9	1.7	<0.001
→Regulation of macromolecule metabolic process	157	91.18	1.72	<0.001
→Regulation of gene expression	114	64.47	1.77	<0.001
Transcription by RNA polymerase II	128	68.59	1.87	<0.001
→Transcription, DNA‐templated	162	93.7	1.73	<0.001
→Cellular macromolecule biosynthetic process	186	118.42	1.57	<0.001
→Metabolic process	460	364.94	1.26	<0.001
→Biosynthetic process	192	123.53	1.55	<0.001
→Macromolecule biosynthetic process	188	120.02	1.57	<0.001
→Organic substance biosynthetic process	192	123.07	1.56	<0.001
Cellular protein modification process	84	50.21	1.67	0.032
→Protein modification process	84	50.36	1.67	0.033
Unclassified	670	820.69	0.82	0
PANTHER GO‐slim cellular component				
Nuclear chromatin	66	36.62	1.8	0.008
→intracellular part	398	311.92	1.28	<0.001
→cell part	540	448.19	1.2	0.001
→cell	544	450.25	1.21	<0.001
Unclassified	809	909.65	0.89	0
Extracellular space	22	56.31	0.39	<0.001
→Extracellular region part	28	62.41	0.45	0.001
→Extracellular region	35	70.2	0.5	0.002
PANTHER pathways				
EGF receptor signaling pathway	29	10.15	2.86	0.001
Cadherin signaling pathway	33	11.98	2.75	<0.001
FGF signaling pathway	24	9.16	2.62	0.015
CCKR signaling map	31	13.2	2.35	0.008
PDGF signaling pathway	26	11.37	2.29	0.046
Wnt signaling pathway	52	23.73	2.19	<0.001
Unclassified	1304	1404.08	0.93	0

**Table 5:** KEGG pathway enrichment analysis of predicted target genes

GOID	GOTerm	Term *P* value^a^	Group *P* value^a^	Nr genes	Associated genes found
KEGG:04520	Adherens junction	<0.001	<0.001	21	ACP1, ACTN4, BAIAP2, CREBBP, CTNND1, EP300, INSR, MAPK1, MAPK3, MET, NLK, SMAD4, SNAI1, SNAI2, SORBS1, SRC, SSX2IP, TGFBR1, VCL, WASL, YES1
KEGG:04110	Cell cycle	<0.001	<0.001	29	CCND2, CDC14A, CDC16, CDC27, CDC6, CDK2, CDKN2B, CDKN2D, CHEK2, CREBBP, CUL1, E2F3, E2F5, EP300, GADD45A, MAD2L1, MCM5, RAD21, RB1, SKP1, SMAD4, SMC1B, SMC3, STAG1, TTK, WEE1, YWHAB, YWHAG, YWHAQ
KEGG:04350	TGF‐beta signaling pathway	0.001	<0.001	21	ACVR1, ACVR1C, BMPR1B, CDKN2B, CREBBP, CUL1, E2F5, EP300, GDF5, ID2, ID4, MAPK1, MAPK3, NODAL, PPP2R1B, RPS6KB1, SKP1, SMAD4, SMAD5, SMAD7, TGFBR1
KEGG:04012	ErbB signaling pathway	0.004	<0.001	20	CAMK2G, ERBB4, EREG, GRB2, HBEGF, KRAS, MAP2K1, MAP2K4, MAPK1, MAPK10, MAPK3, NCK2, PAK2, PAK4, PAK6, PIK3R1, PRKCB, RPS6KB1, SHC3, SRC
KEGG:04360	Axon guidance	0.005	<0.001	32	ABLIM1, ABLIM3, ARHGEF12, BMPR1B, CAMK2G, EPHA5, EPHA7, GNAI1, KRAS, MAPK1, MAPK3, MET, NCK2, NFATC2, NRP1, NTNG1, PAK2, PAK4, PAK6, PIK3R1, PTCH1, ROCK2, RYK, SEMA3C, SEMA4A, SEMA4G, SLIT3, SRC, SRGAP1, SRGAP2, SSH2, UNC5B
KEGG:05211	Renal cell carcinoma	0.009	<0.001	17	CREBBP, EP300, EPAS1, ETS1, GRB2, HIF1A, KRAS, MAP2K1, MAPK1, MAPK3, MET, PAK2, PAK4, PAK6, PIK3R1, RAP1A, SLC2A1
KEGG:04114	Oocyte meiosis	0.011	<0.001	25	ADCY7, AURKA, CAMK2G, CDC16, CDC27, CDK2, CPEB2, CPEB4, CUL1, FBXW11, MAD2L1, MAP2K1, MAPK1, MAPK3, PPP1CB, PPP2R1B, PPP2R5C, RPS6KA3, SKP1, SMC1B, SMC3, SPDYE1, YWHAB, YWHAG, YWHAQ
KEGG:04218	Cellular senescence	0.048	<0.001	28	CCND2, CDK2, CDKN2B, CHEK2, E2F3, E2F5, ETS1, FBXW11, FOXO3, GADD45A, HIPK1, HIPK3, KRAS, LIN52, LIN9, MAP2K1, MAP2K6, MAPK1, MAPK3, NBN, NFATC2, PIK3R1, PPP1CB, RASSF5, RB1, RRAS2, TGFBR1, TSC1

a Corrected with Bonferroni step down.

### 3.9 MiR‐1284 suppressed glioma cell proliferation and migration

**Figure 7:**
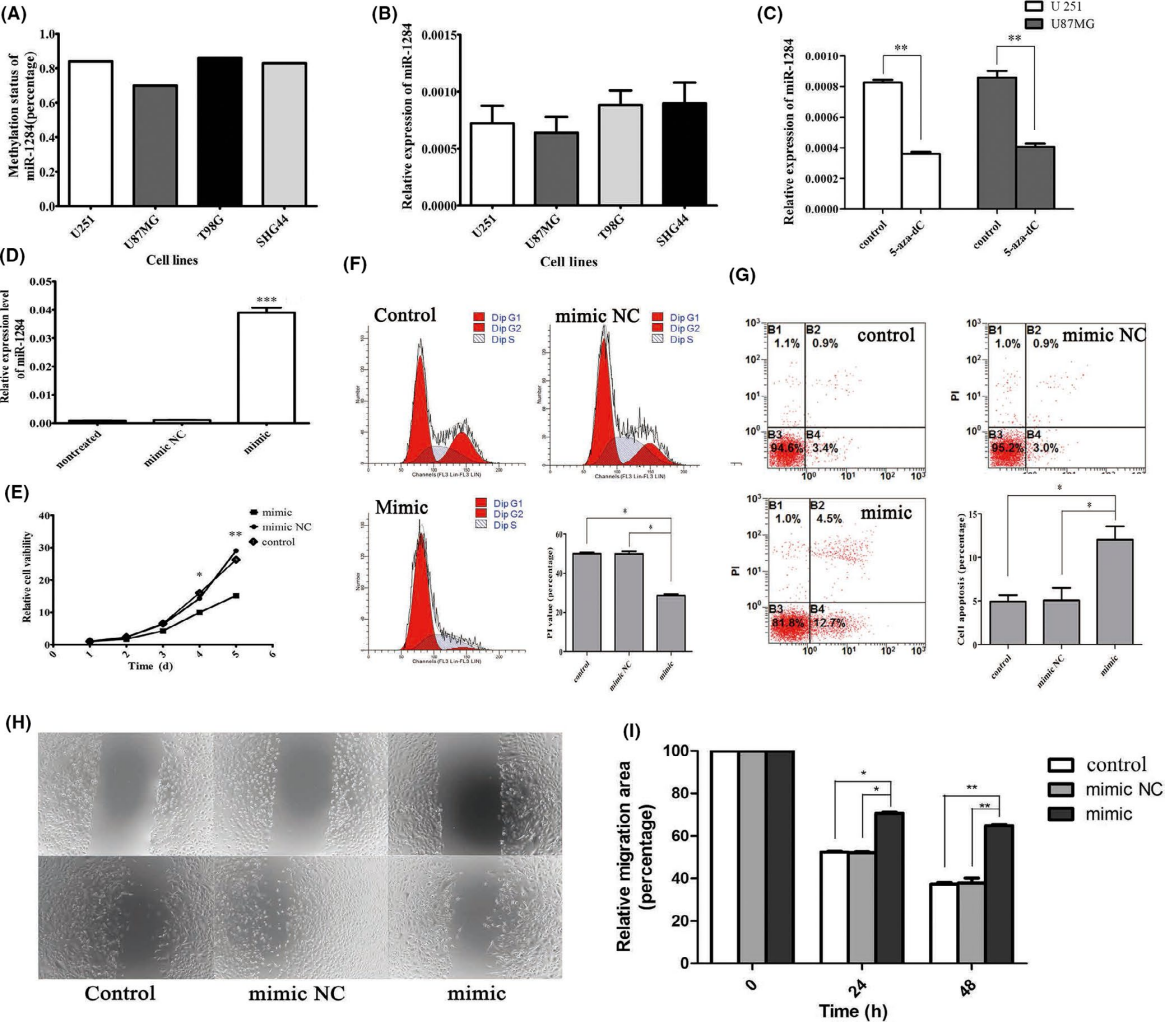
Characteristics of miR‐1284 in GBM cell lines. A, Methylation level of miR‐1284 in glioma cell lines (U251, U87MG, T98G, and SHG44). B, Relative expression of miR‐1284 compared with U6 in glioma cell lines. No difference was found between each cell line. C, Expression of miR‐1284 by qRT‐PCR in U251 and U87MG cells treated with 5‐Aza‐2‐deoxycytidine (AZA). D, Expression of miR‐1284 transfected with mimic and mimic NC for 48 h (*P* < 0.001). E, CCK‐8 assay testing cell viability from 1 to 5 d. F, Flow cytometry detecting cell cycle of U251 and PI values in different groups (G) Flow cytometry testing cell apoptosis after transfection. H, Representative results of wound‐healing assay and the percentage of healing area determined using the ImageJ. *P < 0.05, **P < 0.01, ***P < 0.001

To further validate the functional relevance of this miRNA methylation signature, we selected miR‐1284 for in vitro experiments. Pyrosequencing of cg20382675 showed that the miR‐1284‐associated CpG was consistently associated with high DNA methylation status in glioma cells, that is, U87MG, U251, T98MG, and SHG44 (Figure 7A). Accordingly, the expression levels of miR‐1284 were comparable in those glioma cells (Figure 7B). However, after treated with 5‐Aza‐dC, we found that the expressions of miR‐1284 were significantly decreased in U251 and U87MG, indicating a positive impact of DNA methylation on miRNA expression (Figure 7C). By transferring miR‐1284 mimics into U251, we established a miR‐1284‐overexpressed U251 model, which was validated by qPCR (Figure 7D). CCK‐8 analysis showed that over‐expression of miR‐1284 reduced cell viability of U251 (Figure 7E). Flow cytometry analysis showed that over‐expression of miR‐1284 was also associated with lower frequency of tumor cells in S and G2 phase (Figure 7F), and higher frequency of apoptotic cells (Figure 7G). Finally, wound‐healing assay showed that migration was inhibited by over‐expression of miR‐1284 (Figure 7H).

## 4 DISCUSSIONS

This study investigated genome‐wide DNA methylation microarray data of miRNA‐associated CpGs to explore the clinical value of miRNA methylation in non‐G‐CIMP GBMs. We identified a 5‐CpG signature of miRNA methylation which could predict survival of non‐G‐CIMP GBM patients. This signature showed consistent and robust prognostic values within each subgroup of different ages, molecular subtypes, and treatments and was validated in independent patient cohort. Notably, different risk groups distinguished by this signature showed different bevacizumab therapy outcomes. These findings suggest that methylation status of this 5‐CpG relative miRNAs is closely correlated with GBM malignancies, especially with tumor angiogenesis, and this 5‐CpG methylation signature has good potential to be an indiction for bevacizumab therapy in non‐G‐CIMP GBMs.

As GBMs are characterized by high heterogeneity and massive vessels, anti‐VEGF therapy was expected to improve the outcome of GBM patients.^27^ Bevacizumab, a humanized monoclonal antibody against VEGF, has been the most promising anti‐angiogenic agents for treating GBMs and was approved for recurrent GBM treatment.^28^ However, in newly diagnosed GBM patients, recent randomized trials failed to yield clear survival benefits when applied bevacizumab plus Stupp regimen,^29^ implying that proper indicators are needed for bevacizumab treatment. In this study, the miRNA methylation‐based risk subgroups were associated with differential enrichments of pro‐angiogenic gene sets (eg, hypoxia or VEGF pathways), suggesting the possibility of differential responses to bevacizumab within the risk subgroups. Accordingly, distinct survival benefits were observed in Rennes cohort with the use of bevacizumab at progression: High‐risk patients seemed to benefit more from the bevacizumab‐contained therapy. These results suggest that the 5‐CpG signature is of potential use to optimize bevacizumab therapy by identifying appropriate patient candidates.

For biological features of this 5‐CpG signature, target genes of relative miRNAs were analyzed with bioinformatic methods. Results showed these miRNAs regulated a great many genes and might cooperate with each other to regulate specific genes. The profound characteristics of the target genes were analyzed based on GO database and KEGG database. From the results of GO analysis of BF of these target genes, we can infer that these miRNAs might greatly participate in biological regulation, especially regulation of transcription by RNA polymerase II, also in metabolic processes such as macromolecule biosynthetic process and cellular protein modification process. For MF, these miRNAs might affect ubiquitin‐like protein transferase activity, RNA polymerase II transferase activity, DNA‐binding process, and catalytic activity especially in MAP kinase activity. For CC results, these miRNAs might regulate synthesization of nuclear chromatin and extracellular space components. Pathway enrichment analysis implied these miRNAs were correlated with differentiation, cell motility, immunology, cell proliferation, and migration. Of note, the pathway enrichment analysis showed target genes were enriched in pathways of renal cell carcinoma (KEGG:05211), TGF‐beta signaling pathway (KEGG:04350), and ErbB signaling pathway (KEGG:04012). The renal cell carcinoma pathway includes HIF‐α pathway and strongly correlates with VEGF and PDGF production. This is consistent with the above GSEA results on gene expression data of TCGA and reminds the effects of these signature relative miRNAs.

To further explore the biological relevance of the 5‐CpG signature, we selected one miRNA (miR‐1284) for functional experiments. MiR‐1284 has been reported to have tumor‐inhibiting roles in lung, gastric and ovarian cancers,^30^, ^31^, ^32^ but its roles in GBMs were still unknown. The in vitro experiments confirmed the anti‐tumor role of miR‐1284 as inhibiting glioma cell proliferation and migration and inducing glioma cell apoptosis. Interestingly, when treated with demethylation agent, the expression of miR‐1284 was significantly decreased, which indicated a positive correlation between its DNA methylation and expression. These results supported the biological implications of the 5‐CpG signature that higher methylation status of miR‐1284 was positively correlated with patient survival. In general, hypermethylation of promoter inhibits transcription processes and decreases miRNA expression. However, there have been emerging evidences reporting that DNA methylation could facilitate the expression in some situations despite not knowing the exact mechanisms.^33^, ^34^

Among the other panel‐associated miRNAs, some have been reported to be implicated in glioma biology. MiR‐132 was upregulated in GBMs and was a potential indicator of poor prognosis.^35^, ^36^ MiR‐127 and miR‐433 are both derived from an overlapping gene locus and colocalized within a cancer‐associated genomic region.^37^ MiR‐127 was reported to promote GBM cell migration and invasion by targeting tumor‐suppresser gene SEPT7.^38^ MiR‐433 was reported to be commonly dysregulated in GBMs and suppressed glioma cell proliferation, migration, invasion, and enhanced sensitivity to TMZ therapy.^39^, ^40^ Regarding miR‐759 and miR‐1248, no biological or clinical evidences have been reported in cancers so far.

Our study has several limitations. First, as a retrospective study, the identification and validation of the signature were based on open source databases which had already been uploaded before. The follow‐up information of these researches could not be considered in our study. Also, clinical information such as drug data and recurrent therapy of some cases was not detailed enough, which made it hard to make more subtle analysis. Second, bias caused by the differences among these selected trial platforms should be considered even with compensatory statistical measure. More proof should be collected before conducting further trials. Third, we only performed in vitro study on one miRNA, more in vitro and in vivo studies are needed, especially those on GBM angiogenesis.

In conclusion, by analyzing genome‐wide DNA methylation microarray data of miRNAs‐associated CpGs, we presented the initial report on the prognostic relevance of aberrant DNA methylation in miRNA regions in GBMs. The identification of the biologically and clinically relevant miRNA methylation signature may represent a promising approach for optimizing prognostication of GBMs, and be of potential value for improving individualized treatment and anti‐angiogenic therapy in particular.

## CONFLICT OF INTEREST

No potential conflicts of interest were disclosed.
